# A Genome-First Approach to Estimate Prevalence of Germline Pathogenic Variants and Risk of Pancreatic Cancer in Select Cancer Susceptibility Genes

**DOI:** 10.3390/cancers14133257

**Published:** 2022-07-02

**Authors:** Esteban Astiazaran-Symonds, Jung Kim, Jeremy S. Haley, Sun Young Kim, H. Shanker Rao, Regeneron Genetics Center, David J. Carey, Douglas R. Stewart, Alisa M. Goldstein

**Affiliations:** 1 Clinical Genetics Branch, Division of Cancer Epidemiology and Genetics, National Cancer Institute, NIH, 9609 Medical Center Drive, Rockville, MD 20850, USA; jung.kim2@nih.gov (J.K.); sunyoung.kim@nih.gov (S.Y.K.); drstewart@mail.nih.gov (D.R.S.); 2 Geisinger Clinic, Geisinger Health System, 100 N Academy Ave, Danville, CA 17821, USA; jshaley@geisinger.edu (J.S.H.); hsrao@geisinger.edu (H.S.R.); djcarey@geisinger.edu (D.J.C.); 3 Department of Genetic Medicine, Johns Hopkins University School of Medicine, 733 N Broadway, Baltimore, MD 21205, USA; 4 Regeneron Genetics Center, 777 Old Saw Mill River Rd, Tarrytown, NY 10591, USA; support@regeneron.com

**Keywords:** cancer risk, pancreatic cancer, cancer genetics, pancreatic ductal adenocarcinoma, inherited cancer

## Abstract

**Simple Summary:**

Prevalence and cancer risk estimates derived from the evaluation of affected individuals in the clinic are subject to ascertainment bias. This limitation can be mitigated using a genome-first approach in which genotypic data is analyzed before knowing a patient’s phenotype. Our study aimed to analyze two large cohorts with available exome and phenotype data unselected for a specific diagnosis from a genome-first perspective focusing on six pancreatic cancer predisposition genes. We provide estimates of (1) prevalence of heterozygotes for the general population and for individuals with pancreatic cancer and (2) cancer risk for pancreatic cancer for each gene evaluated. For mutation carriers, we found an elevated risk of pancreatic cancer for most genes evaluated, with variation among genes. This work expands our knowledge of the complex genetics of this cancer and will help identify patients at the highest risk who could benefit from future screening or therapeutic strategies.

**Abstract:**

Patients with germline pathogenic variants (GPV) in cancer predisposition genes are at increased risk of pancreatic ductal adenocarcinoma (PDAC), the most common type of pancreatic cancer. The genes most frequently found to harbor GPV in unselected PDAC cases are *ATM, BRCA1*, *BRCA2*, *CDKN2A*, *CHEK2*, and *PALB2*. However, GPV prevalence and gene-specific associations have not been extensively studied in the general population. To further explore these associations, we analyzed genomic and phenotypic data obtained from the UK Biobank (UKB) and Geisinger MyCode Community Health Initiative (GHS) cohorts comprising 200,600 and 175,449 participants, respectively. We estimated the frequency and calculated relative risks (RRs) of heterozygotes in both cohorts and a subset of individuals with PDAC. The combined frequency of heterozygous carriers of GPV in the general population ranged from 1.22% for *CHEK2* to 0.05% for *CDKN2A*. The frequency of GPV in PDAC cases varied from 2.38% (*ATM)* to 0.19% (*BRCA1* and *CDKN2A)*. The RRs of PDAC were elevated for all genes except for *BRCA1* and varied widely by gene from high (*ATM*) to low (*CHEK2*, *BRCA2*). This work expands our understanding of the frequencies of GPV heterozygous carriers and associations between PDAC and GPV in several important PDAC susceptibility genes.

## 1. Introduction

Pancreatic cancer is one of the deadliest cancers to date, with high mortality despite the improvement in the treatments of cancers in general. This is in part due to the late discovery of pancreatic cancer, which makes treatment and management challenging [1]. Pancreatic ductal adenocarcinoma (PDAC) is the most common form of pancreatic cancer and is associated with the worst prognosis [2].

It has been estimated that 5–10% of PDAC cases have a genetic basis [3,4,5,6]. Many germline pathogenic variants (GPV) in cancer predisposition genes that are part of several inherited cancer syndromes, such as hereditary breast and ovarian cancer syndrome and Lynch syndrome, have been identified in patients with PDAC [3].

A recent systematic review identified *ATM*, *BRCA1*, *BRCA2*, *CDKN2A*, *CHEK2*, and *PALB2* as the most frequent genes harboring GPV in individuals with PDAC not meeting criteria for any cancer predisposition syndrome with varying degrees of family history [4]. However, the risk of PDAC related to GPV, the frequency of GPV carriers in PDAC patients, and the prevalence of PDAC in GPV carriers in these genes is not completely understood. 

Furthermore, much of what is known about the genetics of PDAC has occurred when individuals are evaluated in the clinic due to relevant personal or family history of PDAC and undergo molecular diagnostic testing. While identifying GPV in individuals with PDAC provides a helpful perspective regarding the association of these variants and the risk of developing this cancer, this approach may overestimate associations because of the potential for ascertainment bias. 

One way to mitigate this concern is using a “genome-first” approach. Notably, there is a growing number of biobanks of population-based and health system cohorts with available germline exome data. These projects allow for innovative ways to explore PDAC by the analysis of population genomics data and identification of genetic variation, followed by subsequent abstraction of relevant clinical phenotypes that have been observed. A genome-first approach allows for a more accurate measurement of the prevalence of GPV and risk of developing PDAC in patients with this diagnosis independent of previous personal or family history with reduced ascertainment bias, especially when more than one biobank is analyzed. 

In this study, we analyzed exome data from participants from two databases of population genomics data [UK Biobank (UKB) and the Geisinger MyCode Community Health Initiative (GHS) cohorts] [7,8] to identify GPV, calculate the prevalence of heterozygous carriers, and estimate the risk of developing PDAC for individuals harboring GPV in six important pancreatic cancer susceptibility genes.

## 2. Materials and Methods

We focused on the six genes with the highest frequencies of GPV in individuals with PDAC unselected for family history: *ATM, BRCA1*, *BRCA2*, *CDKN2A*, *CHEK2*, and *PALB2*. We analyzed GPV in these six genes in participants of two large exome databases (UKB and GHS) and evaluated their relationship with pancreatic cancer (Table S1). 

### 2.1. Exome Databases, Sequencing, and Variant Annotation

UKB is a large population-based prospective study in the United Kingdom with adult participants at recruitment, with extensive matching phenotypic and genomic data. The approach used at the Regeneron Genetics Center to perform exome sequencing (ES) in DNA samples from the UKB study, as well as quality metrics and variant filtering, has been previously described in detail [9,10,11]. The present study focused on the 200,600 participants with exome data available in late 2020. The cohort is mostly of European descent (87.7%) and the average age is 69 years. 

Geisinger is an integrated health system in south-central and northeastern Pennsylvania. All Geisinger patients are eligible to participate in the MyCode Community Health Initiative, a system-wide biorepository of blood and DNA samples for broad future research. Participants agree to allow their sample data to be linked to information in their Geisinger health records. MyCode enrollment is open to all patients regardless of the medical conditions they have. MyCode DNA samples were exome sequenced by the Regeneron Genetics Center using IDT exon capture probes as previously described [8]. Coverage depth was sufficient to provide more than 90% coverage of the targeted bases for 99% of samples. Alignments and variant calling were based on the GRCh38 human genome reference sequence. The cohort is mostly of European descent (96.5%) and the average age is 57 years. The Geisinger study population consisted of the first 175,449 MyCode participants to have available exome sequence data. 

Exome and phenotype (PDAC) data from a total of 376,049 participants were included in the study. The exome data were annotated with snpEFF [12] and ANNOVAR [13]. Subsequently, we predicted pathogenicity by ClinVar [14] (data obtained 8 June 2021) and InterVar (version 2.1.3) [15]. Variants determined to be pathogenic or likely pathogenic by these tools were then curated based on the American College of Medical Genetics and Genomics and the Association for Molecular Pathology (ACMG/AMP) [16]. Briefly, variants were evaluated based on allele frequency using The Genome Aggregation Database (gnomAD v2.1.1) (data obtained 10 November 2021), a resource of summarized data from large-scale sequencing projects (https://gnomad.broadinstitute.org/), effect on protein (truncating vs. non-truncating), aggregate in silico predictors from Franklin Genoox (https://franklin.genoox.com/clinical-db/home) (data obtained 15 December 2021), and evidence obtained from functional studies and case-control studies from the literature.

### 2.2. Electronic Health Records (EHR) Review

We used data from the EHR to identify participants with PDAC in the UKB and GHS cohorts. Specifically, clinical phenotypes of PDAC were determined using validated phenotype algorithms that use International Classification Diseases (ICD) diagnosis codes (C25) for PDAC. The code for tumors of the endocrine pancreas (C25.4) was excluded. In addition to longitudinal electronic health record data, we also queried the Geisinger cancer registry, which contains information on all patients diagnosed with cancer at a Geisinger facility; the cancer registry data is also contributed to the National Cancer Database [17]. 

### 2.3. Statistical Analysis 

Prevalences of GPV for each of the six genes were tabulated for the case patients and noncarrier controls in the UKB and GHS, and 95% confidence intervals (CI) were estimated. 2 × 2 contingency tables were then used to test for association of variants and a diagnosis of PDAC by relative risk (RR) and 95% CI. The significance of the association was evaluated using Fisher’s exact test values (*p* < 0.05). 

## 3. Results

### 3.1. Frequency of GPV in Six PDAC Susceptibility Genes in All Participants and in Individuals with PDAC 

First, as the frequency of heterozygous carriers of these GPV in the general population is not well understood, we analyzed GPV in these six genes in the 376,049 participants from both cohorts. Overall, 1.22% of participants carried a GPV in *CHEK2* followed by *ATM* (0.46%), *BRCA2* (0.38%), *PALB2* (0.16%), *BRCA1* (0.15%), and *CDKN2A* (0.05%) (Table 1). No individuals were found to be homozygous for variants in any of the genes evaluated. 

Next, we focused on the individuals in both cohorts who were found to have a diagnosis of PDAC. Table 2 shows the frequency of GPV in these PDAC participants for each of the six genes by cohort, combined and compared with reports from the literature. Overall, 6.94% (5.99% from UKB and 7.60% from GHS) of PDAC patients had a GPV in one of the six genes investigated. Individually, in UKB with 417 reported PDAC patients, 2.64% (*n* = 11) had *ATM* GPV, followed by *CHEK2* (1.44%, *n* = 6) and *BRCA2* (1.20%, *n* = 5). In the GHS cohort with 592 patients with PDAC, 2.70% of PDAC patients had a *CHEK2* GPV followed by *ATM* (2.19%) and *BRCA2* (1.52%).

### 3.2. Prevalence of PDAC in All Participants and in Individuals Harboring GPV in the Six Genes 

**Figure 1:**
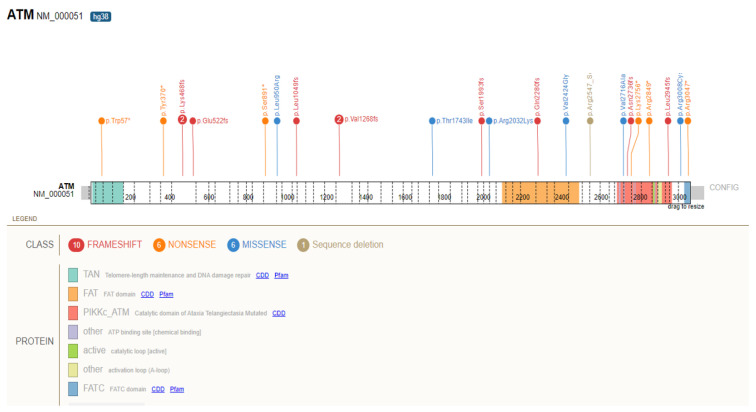
Distribution of germline pathogenic variants in *ATM* identified in participants with pancreatic ductal adenocarcinoma from the UK Biobank and Geisinger MyCode Health Initiative cohorts. Splice site variant (c.8786 + 1G > A) not shown.

**Figure 2:**
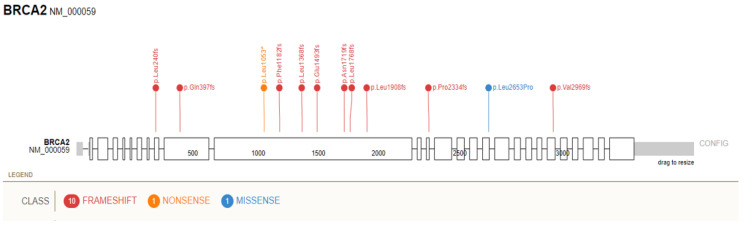
Distribution of germline pathogenic variants in *BRCA2* identified in participants with pancreatic ductal adenocarcinoma from the UK Biobank and Geisinger MyCode Health Initiative cohorts. Splice site variants (c.7977-1G > C and c.8487 + 1G > C) not shown.

The prevalence of individuals with PDAC in UKB was 417 (0.23%) and 592 (0.34%) in the GHS cohort, for a total of 1009 PDAC patients in both cohorts combined (0.27%). Next, to understand the number of patients who developed PDAC and who also carried GPV in the six evaluated genes, we calculated the prevalence of PDAC in this subset of patients (Table 3). Although based on small numbers, the highest prevalence of PDAC in UKB was observed for *CDKN2A* (1.45%) and in the GHS cohort for *PALB2* (2.43%). Across both cohorts combined, 24 individuals with an *ATM* GPV (1.39%), 22 subjects with a *CHEK2* GPV (1.16%), and 14 participants with a *BRCA2* GPV (0.98%) were found to have PDAC. At a variant level, *ATM* (Figure 1) and *BRCA2* (Figure 2) showed the widest spectrum of allelic variation in PDAC participants, with 22 and 14 unique variants, respectively. In contrast, the *CHEK2* variants c.470T>C (p.Ile157Thr) and p.Glu457fs (c.1100delC) were observed in 19 of the 22 GPV heterozygous carriers. The p.Arg170fs variant in *PALB2* was the only *PALB2* GPV detected more than once in PDAC patients, while variants in *BRCA1* and *CDKN2A* were all unique. None of these individuals were related. Supplementary Table S1 presents all the GPV in these six genes identified in patients with PDAC. The median age at cancer diagnosis for individuals with GPV in any gene evaluated was 66.9 for UKB (vs. 65.4 in those without GPV) and 65 for GHS (vs. 66.9 in those without GPV). None of the differences reached statistical significance (*p* < 0.05).

### 3.3. Risk of PDAC in Participants Harboring GPV in ATM, BRCA1, BRCA2, CDKN2A, CHEK2, and PALB2

**Figure 3:**
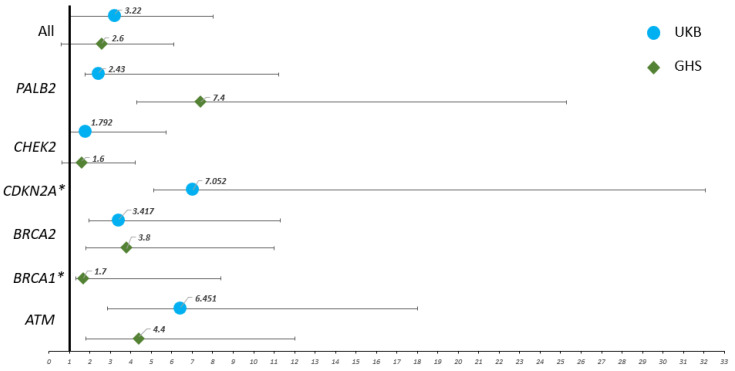
The relative risk of pancreatic ductal adenocarcinoma in participants harboring germline pathogenic variants for each gene. PDAC: pancreatic ductal adenocarcinoma, GPV: germline pathogenic variant. UKB: UK Biobank; GHS: Geisinger MyCode Health Initiative. * Variants identified only in one cohort.

Next, we estimated the RR of PDAC in individuals harboring GPV for each of the six genes in the two cohorts (Table 4 and Figure 3). The highest RR was observed for *CDKN2A* (RR = 7, 95% CI 1.9–25), followed by *ATM* (RR = 6.4, 95% CI 3.6–11.6), and *BRCA2* (RR = 3.4, 95% CI 1.4–7.9) in UKB and *PALB2* (RR = 7.4, 95% CI 3.1–17.9), followed by *ATM* (RR = 4.4, 95% CI 2.6–7.6), *BRCA2* (RR = 3.8, 95% CI 2.0–7.2), and *CHEK2* (RR = 1.6, 95% CI 1.0–2.6) in GHS. These RRs were significantly increased (*p* < 0.05) for *ATM* and *BRCA2* in both UKB and GHS, whereas *CDKN2A*, *CHEK2*, and *PALB2* were each significant in only a single cohort. 

## 4. Discussion

In this study, we used a genome-first approach to analyze genomic and EHR-linked data from 2 large cohorts to identify individuals who harbor GPV in a select group of genes that are frequently detected in PDAC patients (*ATM*, *BRCA1*, *BRCA2*, *CDKN2A*, *CHEK2*, and *PALB2*). We found the highest prevalence of heterozygotes for GPV in *CHEK2*, followed by *ATM* and *BRCA2*. The prevalence of PDAC among GPV carriers showed some variation across the two cohorts, but overall, the prevalence ranged from 0.3% in *BRCA1* to 1.4% in *ATM*. Finally, all genes had a significantly increased RR for either or both cohorts, except for *BRCA1*. 

The prevalence of heterozygous carriers for GPV in the genes evaluated has been estimated in patients with cancer [18,19,20,21], but their prevalences in the general population are less clear. Moreover, some estimates have been variant-specific (founder variants in *BRCA1*, *BRCA2*, and *CHEK2*) [22,23]. Previous studies analyzing data from gnomAD controls v2.1, have reported a prevalence for heterozygous carriers of known GPV in *CHEK2* (0.67%), *BRCA2* (0.35%), *PALB2* (0.1%), and *BRCA1* (0.38%) [24], while the carrier frequency for *ATM* has been calculated at 0.5–1% [25]. The genome-first approach utilized here allowed us to estimate prevalences in a population unselected for a specific diagnosis and, therefore, may more closely resemble the general population. While prevalences for *ATM*, *BRCA1*, *BRCA2,* and *PALB2* in this study are consistent with prevalences observed in the literature, we calculated that as many as 1.22% of individuals in both cohorts unselected for personal or family history of cancer harbored GPV in *CHEK2*. This frequency was mostly driven by the *CHEK2* founder variants c.470T>C (p.Ile157Thr) and p.Glu457fs (c.1100delC), which could explain the higher frequency in the UKB and GHS cohorts. Indeed, higher prevalences for these two variants have been observed in different Northern European populations [26], disproportionally represented in these two cohorts. Lastly, we estimated a prevalence of *CDKN2A* GPV carriers of 0.03–0.07% for the two cohorts. 

Furthermore, although our sample of PDAC patients was relatively small, our estimates of the prevalence of GPV in these participants were mostly consistent with what has been reported in the literature [4]. Of note, we saw some differences in the prevalence of heterozygotes for these GPV in UKB and GHS, which could be in part due to age differences between the two cohorts. Studies have previously discussed the complex genetics of PDAC characterized by a great heterogeneity of genes involved and an overall lower risk when compared to GPV in the same genes for other cancers [4]. Generally, genes and variants have been divided into high-risk genes with a RR ≥ 5.0, moderate-risk genes with a RR 1.5–5.0, and low-risk genes or genetic modifiers with a RR of 1.01–1.5 [27]. Findings in this study suggest that while these six genes increase the risk of developing PDAC, there is wide variability in the degree of risk across genes. Overall, we saw a high risk of PDAC in *ATM* (both cohorts) and in *CDKN2A* (UKB) and *PALB2* (GHS). A moderate risk was seen for *BRCA2* (both cohorts) and *PALB2* (UKB). Low risk was seen for *CHEK2* (both cohorts). The high risk of PDAC in *ATM* heterozygotes has been described before in a multicenter cohort [28]. Interestingly, this finding contrasts with the more moderate risk that has been estimated for breast cancer in this gene [29]. Li et al. recently reported a moderate risk of PDAC for *BRCA1* heterozygotes (2.36 RR = 2.36; 95% CI, 1.51 to 3.68) and for *BRCA2* heterozygotes (RR = 3.34; 95% CI, 2.21 to 5.06) [30]. Of note, although we observed an RR of 1.7 for PDAC in *BRCA1* heterozygotes and the risk in other studies had been calculated to be 2 to 4-fold [31], our results were not statistically significant. For *CHEK2*, a two-fold association has been reported for patients with familial pancreatic cancer in a recent study from Poland, but the association was not seen in unselected PDAC patients or individuals in familial pancreatic cancer families who did not have cancer [32]. In the case of moderate-risk and low-risk genes, there are even more challenges in deriving management recommendations when compared to more well-studied genes (e.g., *BRCA1* and *BRCA2*). For this reason, GPV carriers are generally recommended to undergo individualized management for PDAC in which other genetic (genetic background evidenced by positive family history, variants in other genes, etc.) and non-genetic factors likely play a role [33].

With the increased availability of clinical-grade sequencing, recommendations regarding which individuals should be genetically tested are expanding. The National Cancer Council Network (NCCN) now recommends germline genetic testing for all individuals with PDAC regardless of age (NCCN Guidelines: Genetic/Familial High-Risk Assessment: Breast, Ovarian, and Pancreatic. Version 1.2020)

Another way to evaluate the associations between PDAC and these genes, which is made possible by the genome-first approach, is by examining the prevalence of PDAC in the subset of unselected individuals harboring GPV. Indeed, this approach allowed for the identification of individuals who carried GPV in cancer predisposition genes who did not have a reported diagnosis of PDAC but who could be at increased risk for it. Although other cancers were not evaluated, it is possible that some individuals who carried GPV in a cancer predisposition gene would not have been ascertained through a traditional phenotype-first approach in the clinic due to a lack of personal or family history of cancer. Furthermore, individuals with GPV, especially in high-risk genes, could possibly benefit from innovative technologies in early PDAC screening, including circulating tumor DNA, exosomes in blood, artificial intelligence, metabolomics, and ion mobility spectrometry, which have shown promising prospects [34].

From the genes evaluated here, *BRCA1*, *BRCA2*, and *PALB2* are part of the ACMG Secondary Findings v3.0 list [35] and laboratories are now recommended to report GPV in these genes even in individuals without a personal or family history of cancer when next generation sequencing is performed [36]. Genome-first approaches may provide some preliminary data to assist with decision-making related to secondary findings. 

Recent advances in targeted therapies for a growing list of inherited cancers have revolutionized treatment for these patients. Indeed, the PARP inhibitor rucaparib (Olaparib) is now approved as maintenance therapy for advanced PDAC with GPV in *BRCA1* and *BRCA2*. Furthermore, a recent investigator-initiated, single-arm phase II study assessed its role on advanced PDAC with GPV in *PALB2* and demonstrated that it is a safe and effective therapy for platinum-sensitive cancers, further expanding the role of this drug in patients with inherited cancer [37].

This study had several limitations, one of which was the small sample of individuals with PDAC. For this reason, the examination of risk for some of the genes may have been underpowered. Related, the 95% CI for risk estimates for some genes (*PALB2*, *CDKN2A*) were particularly wide and the results must be interpreted carefully. In addition, although other genetic and nongenetic factors may modify risk, we could not incorporate these factors into the statistical analyses. Further, the use of ES data in our study precluded us from evaluating gross deletions and insertions, which account for up to 11–13% of *BRCA1* and 2–3% of *BRCA2* [38,39], and ~20% of *CDKN2A* [40] GPV. Moreover, although our cohorts might be more similar to the general population than most case-control studies, the GHS cohort is a health-system-based cohort and thus may include a higher proportion of cancer cases than what is seen in the general population. Finally, both cohorts include participants of mostly European ancestry, which might limit the utility of these findings in more diverse populations. Finally, the study relied on available reported phenotypic data for participants in both cohorts. For this reason, it is possible that individuals with PDAC were not included in the study if the specific ICD10 code was not reported.

## 5. Conclusions

In this study, we analyzed EHR-linked exome data from two large cohorts and focused on a group of genes where GPV are frequently identified in individuals with PDAC to explore the complex associations between germline variation and the development of this cancer. We estimated and confirmed the prevalences of heterozygous carriers for these genes. Overall, we found varying degrees of PDAC risk in the genes assessed, from high (*ATM*) to low (*CHEK2*). This work expands our understanding of the frequency of variation in these genes in the general population and the associations between the development of PDAC in individuals harboring GPV in these genes, especially for *ATM*, *CHEK2*, and *PALB2*, where associations are not as well studied as for other genes. The availability of large exome databases is undoubtedly an invaluable new tool that provides another perspective to our understanding of the intricate relations between genetic factors and the development of pancreatic cancer. 

## Figures and Tables

**Table 1:** Number and prevalence of heterozygotes for GPV for each cohort by gene.

	Number and Prevalence (%, in Parentheses) of Heterozygotes for GPV in UKB (*n* = 200,619)	Number and Prevalence (%, in Parentheses) of Heterozygotes for GPV in GHS (*n* = 175,449)	Number and Prevalence (%, in Parentheses) of Heterozygotes for GPV in Both Cohorts (*n* = 376,068)
*ATM*	839 (0.42%)	885 (0.50%)	1724 (0.46%)
*BRCA1*	223 (0.11%)	356 (0.20%)	579 (0.15%)
*BRCA2*	710 (0.35%)	717 (0.40%)	1427 (0.38%)
*CDKN2A*	137 (0.07%)	47 (0.027%)	184 (0.05%)
*CHEK2*	1619 (0.80%)	2987 (1.69%)	4606 (1.22%)
*PALB2*	397 (0.20%)	205 (0.12%)	6022 (0.16%)

GPV: germline pathogenic variant; UKB: UK Biobank; GHS: Geisinger MyCode Health Initiative.

**Table 2:** Frequency of germline pathogenic variants in participants with pancreatic cancer.

	UKB (*n* = 417)		GHS (*n* = 592)		Both Cohorts (*n* = 1009)	Astiazaran-Symonds et al., 2021
	Number of PDAC Participants with GPVs	Frequency of GPVs in PDAC Participants	Number of PDAC Participants with GPVs	Frequency of GPVs in PDAC Participants	Cumulative Frequency	Cumulative Frequency
ALL P/LP	25 *	5.99%	45	7.60%	6.94%	-
*ATM*	11	2.64%	13	2.19%	2.38%	2.52%
*BRCA1*	0	0.0%	2	0.34%	0.19%	0.99%
*BRCA2*	5	1.20%	9	1.52%	1.38%	2.90%
*CDKN2A*	2	0.48%	0	0.0%	0.19%	0.98%
*CHEK2*	6	1.44%	16	2.70%	2.18%	1.15%
*PALB2*	2	0.48%	5	0.84%	0.69%	0.65%

PDAC: Pancreatic ductal adenocarcinoma; GPV: germline pathogenic variant; UKB: UK Biobank; GHS: Geisinger MyCode Health Initiative. * Addition of all individuals (26) and total (25) are not equal due to one individual carrying two variants.

**Table 3:** Prevalence of PDAC in participants harboring a GPV (i.e., heterozygous) by gene.

	UKB Participants Harboring GPVs Who Developed PDAC	GHS Participants Harboring GPVs Who Developed PDAC	Total Number of Heterozygotes for GPVs Who Developed PDAC
*ATM*	11/839 (1.31%)	13/885 (1.47%)	24/1724 (1.39%)
*BRCA1*	0/223 (0%)	2/356 (0.56%)	2/579 (0.34%)
*BRCA2*	5/710 (0.70%)	9/717 (1.25%)	14/1427 (0.98%)
*CDKN2A*	2/137 (1.45%)	0/47 (0%)	2/184 (1.09%)
*CHEK2*	6/1619 (0.37%)	16/2987 (0.53%)	22/4606 (0.48%)
*PALB2*	2/397 (0.50%)	5/205 (2.44%)	7/602 (1.16%)

PDAC: Pancreatic ductal adenocarcinoma; GPV: germline pathogenic variant; UKB: UK Biobank; GHS: Geisinger MyCode Health Initiative.

**Table 4:** The relative risk of pancreatic cancer in participants harboring a germline pathogenic variant for each gene.

	UKB		GHS	
Gene	RR (95% CI	*p*-Value	RR	*p*-Value
*ATM*	6.4 (3.6–11.6)	<0.0001 *	4.4 (2.6–7.6)	<0.0001 *
*BRCA1*	N/A	>0.9999	1.7 (0.4–6.7)	0.3176
*BRCA2*	3.4 (1.4–7.9)	0.0151 *	3.8 (2–7.2)	0.0007 *
*CDKN2A*	7 (1.9–25)	0.0312 *	N/A	>0.9999
*CHEK2*	1.8 (0.8–3.9)	0.1482	1.6 (1–2.6)	0.0411 *
*PALB2*	2.4 (0.7–8.8)	0.1888	7.4 (3.1–17.9)	0.0006 *

PDAC: Pancreatic ductal adenocarcinoma; GPV: germline pathogenic variant; UKB: UK Biobank; GHS: Geisinger MyCode Health Initiative; N/A: not calculable; RR: Relative risk. * *p*-value < 0.05 (statistically significant).

## Data Availability

Data supporting Table 1, Table 2, Table 3 and Table 4, Figure 1, Figure 2 and Figure 3, and Supplementary material are available upon request from the corresponding author.

## Supplementary Materials

The following supporting information can be downloaded at: https://www.mdpi.com/article/10.3390/cancers14133257/s1. Table S1. Germline pathogenic variants in participants with pancreatic cancer by gene and cohort.
