# Supplementary material for: A Genome-First Approach to Estimate Prevalence of Germline Pathogenic Variants and Risk of Pancreatic Cancer in Select Cancer Susceptibility Genes
Source: Cancers (Basel). 2022 Jul 2;14(13):3257. doi: 10.3390/cancers14133257 (PMC9265005; doi:10.3390/cancers14133257)
Supplement: Supplementary file 1 [file cancers-14-03257-s001.zip › cancers-1784616-supplementary.pdf]

Supplementary Table S1. Germline pathogenic variants in participants with pancreatic cancer by gene and cohort

Gene	DNA change	Amino Acid change	Variant Type	Transcript	Number of participants with GPV in UKB	Number of participants with GPV in GHS
ATM	c.1110C>G	p.Tyr370*	Nonsense	NM_000051.4	0	1
ATM	c.1402_1403delAA	p.Lys468fs	Frameshift	NM_000051.4	0	2
ATM	c.1564_1565delGA	p.Glu522fs	Frameshift	NM_000051.4	0	1
ATM	c.170G>A	p.Trp57*	Nonsense	NM_000051.4	1	0
ATM	c.2672C>G	p.Ser891*	Nonsense	NM_000051.4	0	1
ATM	c.2849T>G	p.Leu950Arg	Missense	NM_000051.4	1	0
ATM	c.3146dupT	p.Leu1049fs	Frameshift	NM_000051.4	1	0
ATM	c.3802delG	p.Val1268fs	Frameshift	NM_000051.4	2	0
ATM	c.5228C>T	p.Thr1743Ile	Missense	NM_000051.4	1	0
ATM	c.5979_5983delTAAAG	p.Ser1993fs	Frameshift	NM_000051.4	0	1
ATM	c.6095G>A	p.Arg2032Lys	Missense	NM_000051.4	0	1
ATM	c.6839delA	p.Gln2280fs	Frameshift	NM_000051.4	0	1
ATM	c.7271T>G	p.Val2424Gly	Missense	NM_000051.4	0	1
ATM	c.7638_7646delTAGAATTTC	p.Arg2547_Ser2549del	In-frame deletion	NM_000051.4	1	0
ATM	c.8147T>C	p.Val2716Ala	Missense	NM_000051.4	0	1
ATM	c.8204_8205dupGT	p.Asn2736fs	Frameshift	NM_000051.4	1	0
ATM	c.8266A>T	p.Lys2756*	Nonsense	NM_000051.4	0	1
ATM	c.8545C>T	p.Arg2849*	Nonsense	NM_000051.4	0	1
ATM	c.8786+1G>A	-	Splicing	NM_000051.4	1	0
ATM	c.8833_8834delCT	p.Leu2945fs	Frameshift	NM_000051.4	1	0
ATM	c.9022C>T	p.Arg3008Cys	Missense	NM_000051.4	0	1
ATM	c.9139C>T	p.Arg3047*	Nonsense	NM_000051.4	1	0
BRCA1	c.5329dupC	p.Gln1777fs	Frameshift	NM_007294.4	0	1
BRCA1	c.68_69delAG	p.Glu23fs	Frameshift	NM_007294.4	0	1
BRCA2	c.1189_1190insTTAG	p.Gln397fs	Frameshift	NM_000059.4	0	1
BRCA2	c.3158T>G	p.Leu1053*	Nonsense	NM_000059.4	1	0
BRCA2	c.3545_3546delTT	p.Phe1182fs	Frameshift	NM_000059.4	0	1
BRCA2	c.4103delT	p.Leu1368fs	Frameshift	NM_000059.4	0	1
BRCA2	c.4478_4481delAAAG	p.Glu1493fs	Frameshift	NM_000059.4	1	0
BRCA2	c.5157_5161delTTCAA	p.Asn1719fs	Frameshift	NM_000059.4	0	1
BRCA2	c.5303_5304delTT	p.Leu1768fs	Frameshift	NM_000059.4	1	0
BRCA2	c.5722_5723delCT	p.Leu1908fs	Frameshift	NM_000059.4	0	1
BRCA2	c.6998dupT	p.Pro2334fs	Frameshift	NM_000059.4	0	1
BRCA2	c.718_719delCT	p.Leu240fs	Frameshift	NM_000059.4	0	1
BRCA2	c.7958T>C	p.Leu2653Pro	Missense	NM_000059.4	1	0
BRCA2	c.7977-1G>C	-	Splicing	NM_000059.4	0	1
BRCA2	c.8487+1G>C	-	Splicing	NM_000059.4	1	0
BRCA2	c.8904delC	p.Val2969fs	Frameshift	NM_000059.4	0	1
CDKN2A	c.104G>C	p.Gly35Ala	Missense	NM_000077.5	1	0
CDKN2A	c.251A>C	p.Asp84Ala	Missense	NM_000077.5	1	0
CHEK2	c.1100delC	p.Thr367fs	Frameshift	NM_007194.4	5	3
CHEK2	c.1368dupA	p.Glu457fs	Frameshift	NM_007194.4	0	1
CHEK2	c.409C>T	p.Arg137*	Nonsense	NM_007194.4	0	1
CHEK2	c.470T>C	p.Ile157Thr	Missense	NM_007194.4	1	10
CHEK2	c.483_485delAGA	p.Glu161del	In-frame deletion	NM_007194.4	0	1
PALB2	c.2712G>A	p.Trp904*	Nonsense	NM_024675.4	0	1
PALB2	c.2727_2728delTT	p.Thr911fs	Frameshift	NM_024675.4	0	1
PALB2	c.2915delT	p.Leu972fs	Frameshift	NM_024675.4	1	0
PALB2	c.3116delA	p.Asn1039fs	Frameshift	NM_024675.4	1	0
PALB2	c.3256delC	p.Arg1086fs	Frameshift	NM_024675.4	0	1
PALB2	c.509_510delGA	p.Arg170fs	Frameshift	NM_024675.4	0	2

Abbreviations: PDAC: pancreatic ductal adenocarcinoma, GPV: germline pathogenic variant. UKB: UK Biobank; GHS: Geisinger MyCode Health Initiative.
